# Factors that influence the caste ratio in a bacterial division of labour

**DOI:** 10.1098/rstb.2023.0267

**Published:** 2025-03-20

**Authors:** Luis Alfredo Avitia Domínguez, Zhengzhou Yu, Varun Chopra, Ruth Viveros, Natalia Tschowri, Roeland Merks, Bram van Dijk, Daniel Rozen

**Affiliations:** ^1^ Institute of Biology, Leiden University, Leiden 2333 BE, The Netherlands; ^2^ Leibniz University, Hannover 30167, Germany; ^3^ Mathematical Institute, Leiden University, Leiden 2333 CC, The Netherlands; ^4^ Department of Biology, Utrecht University, Utrecht 3584 CH, The Netherlands

**Keywords:** mutation frequency, division of labour, *Streptomyces*, genome instability

## Abstract

Colonies of the bacterim *Streptomyces coelicolor* divide labour between cells that specialize in growth and sporulation and cells that specialize in antibiotic production. This division of labour arises owing to costly chromosome deletions in the antibiotic overproducers. However, the spatial distribution and temporal emergence of these mutations in *S. coelicolor* colonies remain unknown, or whether mutation frequency—which we liken to the caste ratio in social insects—is phenotypically plastic. To elucidate changes in the proportions of specialized cells (measured as the mutation frequency), we sampled *S. coelicolor* colonies grown under different conditions. Temporally, mutation frequency increased linearly with colony age and size. Spatially, mutations accumulated disproportionately in the colony centre, despite greater growth and sporulation at the periphery. Exposing colonies to sub-inhibitory concentrations of some antibiotics, a competitive cue in *Streptomyces*, increased mutation frequencies. Finally, direct competition with other *Streptomyces* that naturally produce antibiotics increased mutation frequencies, while also increasing spore production. Our findings provide insights into the intrinsic and environmental factors driving division of labour in *Streptomyces* colonies by showing that mutation frequencies are dynamic and responsive to the competitive environment. These results show that chromosome deletions are phenotypically plastic and suggest that *Streptomyces* can flexibly adjust their caste ratio.

This article is part of the theme issue ‘Division of labour as key driver of social evolution’.

## 1. Introduction

One of the hallmark features of multicellular organisms and complex societies is the division of labour among cells and individuals [1,2]. In multicellular species, this leads to distinct cell types within the body that perform different specialized functions [3]. Analogously, social animal groups divide into different specialized castes that perform complementary functions and work together to increase colony efficiency [4]. In both cases, an assumption is that the proportion of cells or individuals performing specialized tasks is optimized to maximize group fitness [4–6]. This predicts that the caste ratio, measured as the level of investment into different castes or specialized cell types, is not fixed but instead should vary according to the demands of the local environment.

Flexible caste ratios have been observed in several systems. Classic experiments with ants [7] and polyembryonic wasps [8] found that more soldiers were produced when insect colonies were exposed to higher levels of interspecific competition, and moreover that these shifts were adaptive. Similar results were recently reported in parasitic trematode worms that colonize snails as part of their complex life cycle [9,10]. As with ants and wasps, worms that developed in the presence of competition from other parasite species invested more heavily in defensive castes. This result supported the idea of flexible caste ratios, while also broadening the range of species displaying this type of phenotypic plasticity. Our aim in this paper is to explore whether similar principles—that is to say, phenotypically plastic caste ratios—are also evident in multicellular bacteria via plasticity in the generation of specialized cell types.

*Streptomyces* are a group of multicellular bacteria with a complex life cycle [11,12]. Beginning as spores, colonies grow as an interconnected network of branching hyphae called the vegetative mycelium. When resources are exhausted, part of the colony differentiates to produce aerial hyphae that extend above the colony surface and ultimately give rise to a new generation of durable spores. This transition typically coincides with the production of secondary metabolites, including a diverse array of antibiotics [13,14]. *Streptomyces* are responsible for the majority of antibiotics used in medicine and agriculture, in addition to an increasing variety of other compounds and enzymes [13,15].

The partition of the colony into vegetative and aerial hyphae results in a division of labour between growth and reproduction, respectively, that is equivalent to the germ–soma division in eukaryotes [11,16]. We recently discovered a second type of division of labour, which acts solely within the vegetative hyphal population [17]. While most cells contribute to resource acquisition and growth, a minority of cells are responsible for producing metabolically expensive antibiotics [17,18]. An especially surprising aspect of this division of labour is the mechanism that drives it. In contrast to most bacteria, *Streptomyces* contain linear, rather than circular, genomes. It has long been known that these linear chromosomes are highly unstable, resulting in large deletions at the ends of the left and right chromosome arms [19–22]. Deletions can be up to 1 Mb and are highly costly for the cells carrying them [18,19]. However, in the model species we study, *Streptomyces coelicolor*, strains with deletions hyper-produce a diversified set of antibiotics that these bacteria use to compete for food and space [18]. Through this mutation-driven division of labour [23], colonies can maximize both the production of spores and the production of antibiotics.

Although our previous work has clarified some of the costs and benefits of this bacterial division of labour, we know very little about the factors that give rise to this specialized group of antibiotic hyper-producing cells. Hereafter, in analogy with social insects, we refer to these specialized cells as a caste. Specifically, it is currently unknown (i) when and where cells containing genome deletions arise within colonies during growth, (ii) what factors regulate the fraction of cells containing such deletions, and (iii) whether this fraction, i.e. the caste ratio, can respond flexibly to competition. Because *Streptomyces* use secreted antibiotics to compete with each other [24–27], we measured mutation frequencies after exposure to antibiotics and then followed this with head-to-head competition experiments against species that naturally produce the same antibiotics. While antibiotic exposure is an indirect cue for competition [26,28,29], exposure to other species measures the direct response to both resource and antibiotic stress from a growing competitor. Briefly, we find that mutation frequencies increase as a function of colony size and age, while they disproportionately increase in the colony centre. This result contrasts with predictions of ‘allele surfing’ [30,31], which suggests that mutations are more likely to fix at the colony edge owing to increased resource access and growth. In addition, we observe significant changes in mutation frequencies in response to some antibiotics as well as a direct increase following exposure to competitors. These results reveal extensive plasticity in mutation frequencies and suggest that shifting the caste ratio can provide benefits when colonies face competitors.

## 2. Methods

### (a) Bacterial strains and growth conditions

Three *Streptomyces* species were used in this study: *Streptomyces coelicolor* A3(2) (strain M145), *Streptomyces ardus* (DSM 110 40603) and *Streptomyces venezuelae* (DSM 112328). All strains were cultivated on soy flour mannitol medium agar plates (SFM) agar plates at 30°C. SFM contains (per litre): mannitol (20 g), agar (20 g) and soy flour (20 g). Spore stocks were prepared using standard protocols [32] and spore titres were determined by serial dilution.

### (b) Mutation frequency through time

To study the temporal dynamics of mutations in *S. coelicolor,* we diluted our frozen spore stocks to a concentration of roughly 50−100 spores per ml and then plated 100 µl on 80 separate SFM plates. This approach ensured that each plate contained 5−10 well isolated colonies whose growth was unimpeded by crowding. At nine different time points post-inoculation (72, 120, 168, 216, 264, 308, 360, 480 and 648 h), we randomly sampled 16 well isolated colonies to determine number of colony-forming units (CFU), mutant frequency and colony size, for a total of 144 colonies. Colonies were destructively sampled by removing the entire biomass using a sterile loop and transferring it to 400 µl of 30% glycerol. Colonies with obviously aberrant morphology at the first three time points were excluded from sampling to avoid strains derived from mutated spores in the original spore stock.

To determine the area of each colony, pictures of the colonies were taken with a ZEISS AXIO Zoom V16 microscope for the first seven time points, and with a Nikon D200 camera for the last two time points owing to the larger colony size. Colony area was measured in ImageJ v. 1.54d, in which the dimensions of the colony were obtained based on pixel count.

Spores from sampled colonies were collected using a custom microfilter consisting of a cotton wool-plugged 200 μl pipette tip inserted into a hole drilled into the lid of a sterile 1.5 ml centrifuge tube. Pre-assembled filters were covered in aluminum foil and autoclaved. Each colony was vortexed at maximum speed, after which 200 μl from the 30% glycerol solution was pipetted into the cotton-plugged pipette tip. Next, the microfilters were centrifuged at a speed of 12 000 r.p.m. for 1.5 min, which allowed spores and some small mycelial fragments to pass through while trapping most mycelia in the cotton. The process was finally repeated with the remaining volume (approx. 200 μl) from the original collection tube.

CFU was determined for each colony using serial dilution onto SFM agar, after which plates were incubated for 4–7 days at 30°C. To ensure counts and mutation frequencies were obtained from well dispersed colonies, we only used plates with 100−300 total colonies. Mutation frequency was estimated from the same plates by counting colonies with aberrant colony morphologies when compared with the wild-type strain. Mutant colonies had different coloration (indicative of altered antibiotic production or spore pigmentation), irregular shapes, notably smaller sizes and/or no aerial hyphae development in part or all of the colony (electronic supplementary material, figure S1). Putative mutants were independently scored by two people (L.A.A.D. and Z.Y.) and analysed on the fourth day after sample inoculation. Mutation frequencies were quantified by dividing the number of mutants by the total CFU.

### (c) Spatial differences in mutant frequency

To assay the spatial distribution of mutants, colonies were plated as above onto SFM agar to ensure that there were roughly 5−10 well isolated colonies per plate. Colonies (*n* = 32 per time point) were sampled on Day 7 and Day 14 using the same custom microfilter as described above. However, rather than sampling the entire colony, we instead partitioned colonies into an inner and outer region (figure 2). On Day 7, the interior of the colony was sampled by pushing the top of a cut sterile p200 pipette tip through the centre of the colony and then depositing the biomass into a 1.5 ml centrifuge tube containing 400 μl of 30% glycerol, as above. The pipette points were cut before autoclaving to have a diameter of 3 mm. The same procedure was used on Day 14 but with a larger p1000 tip cut to a diameter of 6 mm. The outside of the colony for both time points was sampled by removing all biomass with a loop and treating as above. No part of the colony was left behind, and pairwise data for each colony were obtained. The size of the interior sampled region at both time points corresponded to one-half the radius of the whole colony, leading to a roughly fourfold difference in biomass area. In total, the area of the Day 7 colony corresponded to the interior area of the Day 14 colony, allowing a direct comparison between the two time points. CFU and mutant frequency were scored using the same procedure as above.

### (d) Effects of antibiotics on mutant frequency

To examine whether mutation frequency varied in response to antibiotic exposure, 160 colonies of *S. coelicolor* were grown in the presence of sub-minimal inhibitory concentrations (sub-MICs) of chloramphenicol, erythromycin, gentamycin, kanamycin, streptomycin, tetracycline, rifampicin, mitomycin and novobiocin, using 16 colonies per treatment. Antibiotics were chosen based on prior experiments from our own preliminary studies and work from other laboratories and also to include antibiotics with different modes of action. MIC for each antibiotic (table 1) was determined by plating a spot of 10^5^ spores on plates with different antibiotic concentrations. MIC was scored as the lowest antibiotic concentration where no growth was observed. Mutation frequencies were assayed at 25% of the MIC for all drugs except for streptomycin, where we used 10% of the MIC to avoid effects on viability. The MIC and test concentration for each antibiotic are shown in table 1. Mutation frequencies and CFU for each colony were determined as described above. Chloramphenicol, tetracylcine, streptomycin sulfate, rifampicin, kanamycin monosulfate and erythromycin were obtained from Sigma-Aldrich, novobiocin and mitomycin C were obtained from Cayman Chemical, and gentamycin sulfate was obtained from Duchefa. Antibiotics were prepared in water or 70% EtOH (chloramphenicol, rifampicin, eythromycin and tetracycline) and then filter-sterilized.

**Table 1.** Minimal inhibitory concentrations (MICs) of different antibiotics to *Streptomyces coelicolor* growing on soy flour mannitol medium (SFM) agar. CMP, chloramphenicol; ERY, erythromycin; GEN, gentamycin; KAN, kanamycin; STR, streptomycin; TET, tetracycline; RMP, rifampicin; MMC, mitomycin C; NB, novobiocin.

	antibiotic								
	CMP	ERY	GEN	KAN	STR	TET	RMP	MMC	NB
MIC (μg ml^−1^)	42	8	10	5	2	30	10	2	3
concentration used (μg ml^−1^)	10.5	2	2.5	1.25	0.2	7.5	2.5	0.5	0.75

### (e) Effects of interspecific competition on mutant frequency and number of colony-forming units

To test if mutation frequencies were increased owing to interactions with competitors, we established co-culture experiments between *S. coelicolor* and two other species, *S. venezuelae* and *S. ardus*. Both species naturally produce antibiotics that increase mutation frequencies (figure 3). *Streptomyces venezuelae* produces chloramphenicol, while *S. ardus* produces mitomycin C. In the co-culture, the focal strain, *S. coelicolor,* and competitor strains, *S. ardus* and *S. venezuelae,* were streaked separately on SFM agar along the opposite edges of 1 cm × 1 cm wells of a 25-well plate. The concentration of the spores was adjusted so that there were approximately 20−30 competitor colonies and two to four *S. coelicolor* colonies. The distance between competitors was approximately 8 mm. To test if increased antibiotic secretion from the competitor would further increase the mutation frequency, colonies were plated simultaneously or with a 2 day delay to allow accumulation of antibiotics produced by competitors. Well separated *S. coelicolor* colonies were sampled after 7 days of growth using the protocol above.

### (f) Statistical analysis

Statistical analysis was performed using the *stats* package of R studio software v. 4.0.4, and *ggplot* v. 3.3.5, *ggpubr* v. 0.4.0 and *tidyverse* v. 1.3.1 packages were used to generate graphs [33]. To determine temporal dynamics of mutations we plotted colony area or age versus mutation frequencies and CFU for 144 colonies across time or area. Pearson correlation and linear regression were used to test the relationship between mutation frequency and time, mutation frequency and colony area, and area against CFU. Differences in mutation frequencies and CFU from the interior and exterior of *S. coelicolor* colonies were tested using Wilcoxin signed-rank tests (*V*) to reflect the paired values from each colony. Mann–Whitney *U*-tests were used to compare mutation frequencies and CFU between both time points. This was done in two ways: first by comparing the mutation frequencies and CFU of one time point against the other and second by quantifying total CFU and mutation frequency for the whole Day 7 colonies and comparing them with the values from the interior of Day 14 colonies (reflecting their comparable area). To test for proportional changes in mutation frequency or CFU in the interior versus exterior of the colony, we calculated the ratio of these values and tested for significance using a one-sample Wilcoxon signed-rank test (*W*) with a *µ*-value of 1. To measure the effect of antibiotics on mutation frequencies we used Mann–Whitney *U*-tests with the Benjamini–Hochberg procedure with a *q*-value of 0.05 to control for the false discovery rate. The estimates of change of *S. coelicolor* CFU and mutation frequencies during competition were tested versus the control (*S. coelicolor* versus *S. coelicolor*) using Mann–Whitney *U*-tests.

## 3. Results

### (a) Temporal dynamics of mutation

**Figure 1.**
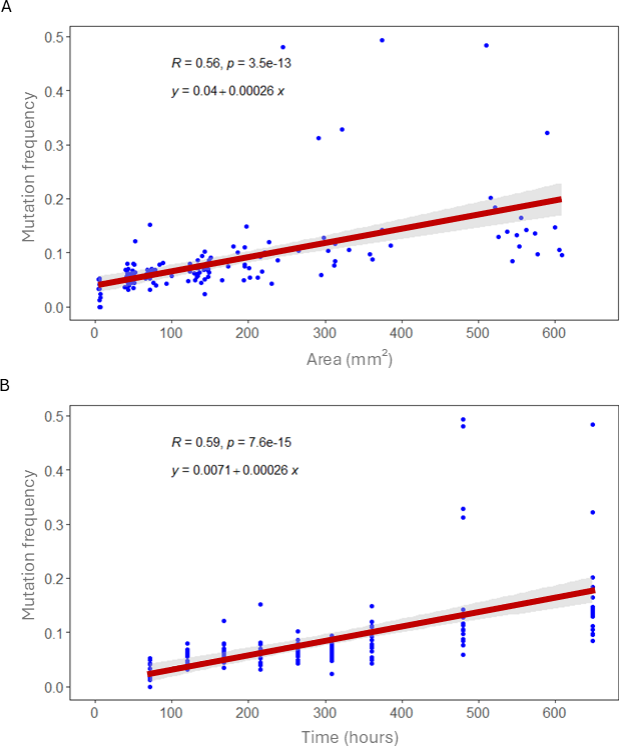
Mutation frequencies are plotted against colony area (*a*) and over time (*b*) for 144 colonies destructively sampled at nine different time points. Pearson correlation and regression analysis are shown in each graph. We found significant linear increases in mutation rate as colonies age and with colony area.

To track changes in mutation frequency through time, we sampled a total of 144 colonies taken over 9 different time points after growth on SFM agar plates. Sixteen colonies were destructively sampled at each time point by removing the entire colony biomass. Our results in figure 1 show that mutation frequency increases significantly as colonies grow and age (area: *R* = 0.56, *p* < 0.001; age: *R* = 0.59, *p* < 0.001). In extreme cases, mutation frequencies can approach 50%. As expected, both colony area and CFU increased continuously through time and are significantly correlated (*R* = 0.78, *p* < 0.001; electronic supplementary material, figure S2).

### (b) Spatial dynamics of mutation

**Figure 2.**
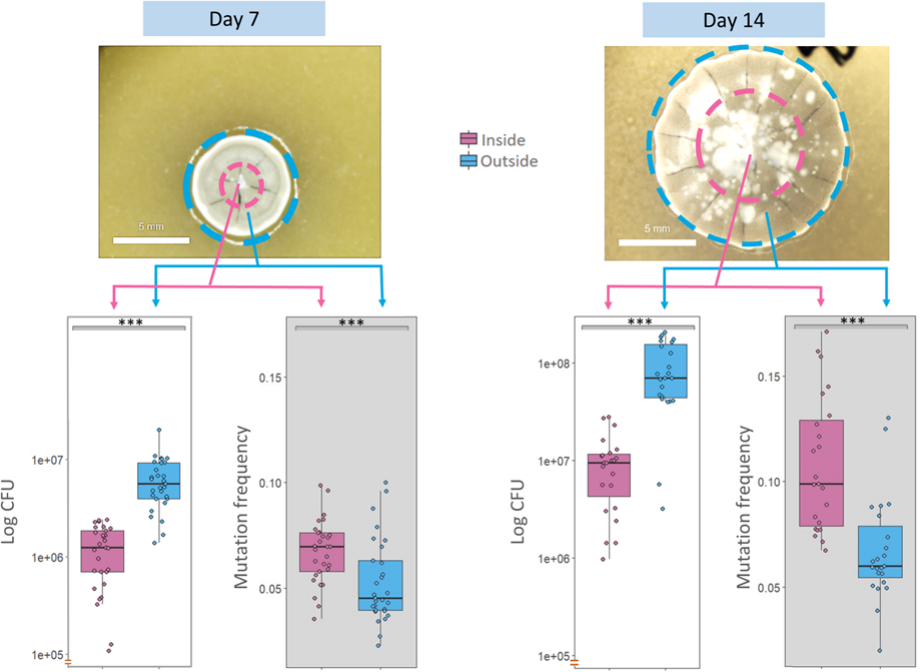
Spatial distribution of mutation frequency and colony-forming units (CFU) in different regions of *Streptomyces* colonies sampled after 7 and 14 days of growth (*n* = 32 colonies per time point). Colonies were sampled from the interior, bounded by a pink circle, and exterior, shown in blue. CFU is shown in white-backgound plots while mutation frequency from each time point is shown on grey. For both time points, mutation frequencies are significantly higher in the colony interior while CFU is higher on the outer edge of the colony. Wilcoxon signed-rank tests were used for significance testing: ***p < 0.001.

Data and theory suggest that mutations that arise at the colony edge, where most growth occurs owing to increased resource access, are more likely to fix via the process of ‘allele surfing’ [30,31]. This idea predicts that mutation frequencies would be higher at the colony exterior when compared with the inside of the colony. To test if results in figure 1 could be explained by this possibility we quantified mutation frequencies and CFU from the centre and the edge of 7- and 14-day-old colonies (figure 2). As expected, CFU and mutation frequency both increased from Day 7 to Day 14 (CFU: *U* = 34, *p* < 0.001 ; mutation frequency: *U* = 52, *p* < 0.001). While the CFU and mutation frequency on Day 7 were approximately 9 × 10^6^ and approximately 0.06 on average, they were 1 × 10^8^ and approximately 0.09 on Day 14. Looking more closely, however, we observed striking differences as a function of where spores were sampled. CFU between Day 7 and Day 14 increased almost exclusively at the colony edge (figure 2, white-background plots). That is to say, in the interior, the spore numbers in Day 14 colonies did not differ from the number of spores in Day 7 colonies (*U* = 277, *p* = 0.305), while we observed a significant CFU increase on the colony edge (*U* = 45, *p* < 0.001). The situation is reversed for mutation frequency. While the mutation frequency on the inside of the colony was significantly increased in Day 14 colonies (*U* = 45, *p* < 0.001), the outside remained unchanged (*U* = 256, *p* = 0.15; figure 2, grey-backgound plots). On both Days 7 and 14, the mutation frequency was significantly higher in the inside versus the outside of the colony (paired Wilcoxon signed-rank test: Day 7: *V* = 361, *p* = 0.001; Day 14: *V* = 263, *p* < 0.001). However, there were no significant differences in the ratio of interior : exterior across time points (*W* = 262, *p* = 0.19). Thus, although our data showed evidence of task division between the interior and the exterior of *Streptomyces* colonies, the spatial location of the castes was the reverse of predictions based on the idea of allele surfing.

**Figure 3.**
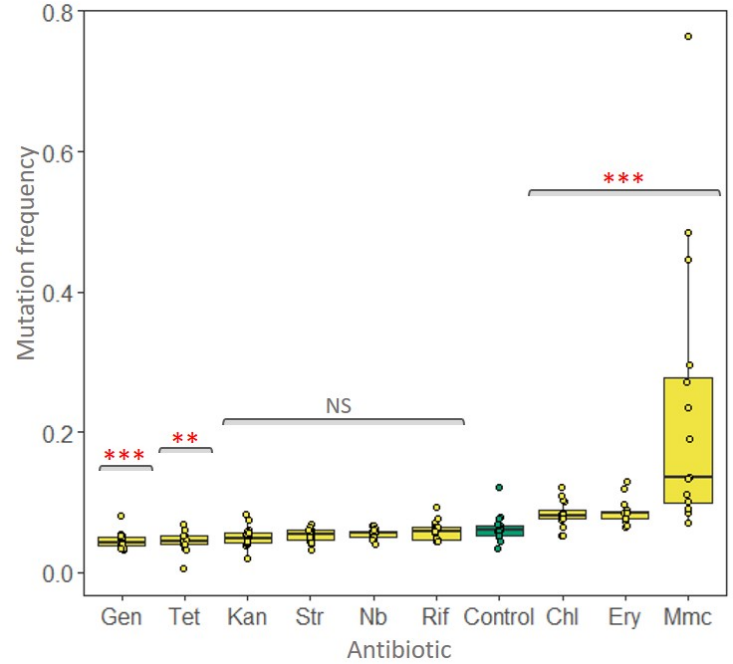
Mutation frequencies of *Streptomyces coelicolor* grown in the presence of one-quarter minimal inhibitory concentrations of different antibiotics (see table 1 for concentrations). Tetracycline (Tet), gentamicin (Gen), kanamycin (Kan), streptomycin (Str), noboviocin (Nb), rifampycin (Rif), erythromycin (Ery) and mitamycin (Mmc) were used. Control colonies grown without antibiotics are shown in green. For significance testing, Mann–Whitney *U*-tests were used with Benjamini–Hochberg correction of 5%. NS, not significant, **p < 0.01, ***p < 0.001.

### (c) Antibiotic responsiveness

To determine if mutation frequency was influenced by external conditions, i.e. to test if external conditions could give rise to shifts in caste ratios, we exposed colonies of *S. coelicolor* to sub-MICs of a set of diverse antibiotics. Bacteria were plated on SFM agar supplemented with antibiotics at low densities to ensure that colonies did not influence the growth of neighbours. Antibiotics are ecologically relevant cues in *Streptomyces* as these species are prolific producers of these anti-competitor toxins. In addition, several studies have shown that sub-MICs of antibiotics select for antibiotic resistance and can induce antibiotic production [26,28,29]. The mutation rate of the control sample without antibiotics was 0.056 (range: 0.041–0.073.) Of nine tested compounds, five caused mutation frequencies that were significantly different from what we observed on drug-free agar (figure 3). Of these, chloramphenicol, erythromycin and mitomycin C gave higher frequencies than the control, with mean values of 0.083 (range: 0.052–0.121), 0.087 (range: 0.065–0.129) and 0.23 (range: 0.07–0.763), respectively, and two, gentamycin and tetracycline, gave lower than the control, with mean values of 0.045 (range: 0.033–0.067) and 0.044 (range: 0.031–0.054), respectively. We observed no difference in mutation frequencies for cells exposed to kanamycin, streptomycin, rifampicin or novobiocin (see figure 1 legend for statistical details). These results show that environmental cues (antibiotics) that are naturally produced by competing *Streptomyces* species can significantly modify the caste ratio in *S. coelicolor* colonies.

### (d) Flexible caste ratios in response to competition

As shown above, several antibiotics can cause an increased mutation frequency when colonies are grown on plates containing sub-MICs of antibiotics. However, the antibiotic concentrations we used during these assays are not necessarily reflective of the concentrations *Streptomyces* colonies experience during direct interspecific competition. We, therefore, asked if direct competition with *S. venezuelae* and *S. ardus* which naturally produce chloramphenicol and mitomycin C, respectively, sufficed to induce mutations in *S. coelicolor*. Both antibiotics caused an increased mutation frequency in *S. coelicolor* (figure 3).

**Figure 4.**
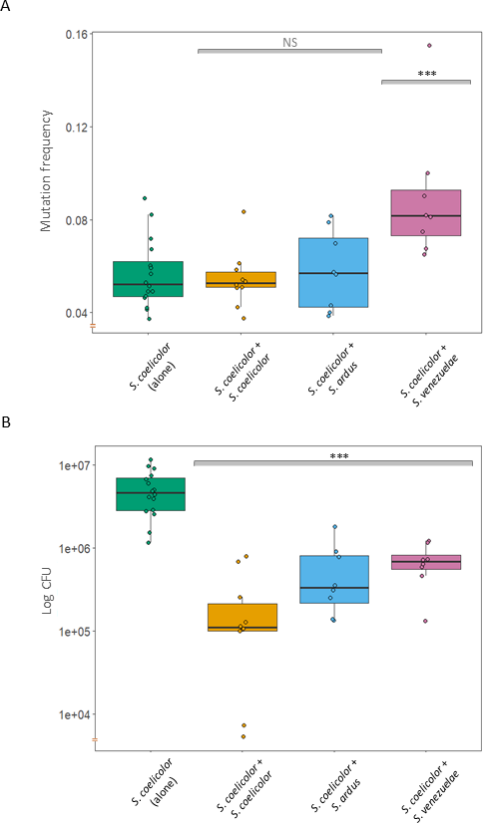
Mutation frequency (*a*) and number of colony-forming units (CFU) (*b*) after *Streptomyces coelicolor* was grown together with *Streptomyces venezuelae* or *Streptomyces ardus* (and versus self). *Streptomyces venezuelae* naturally produces chloramphenicol and *S. ardus* naturally produces mitomycin C. Control treatments correspond to *S. coelicolor* grown in the absence of a competitor (alone). Mann–Whitney *U*-tests were used for significance testing. NS, not significant; ***p < 0.001.

As shown in figure 4A, in pairwise competition assays we found no difference between the mutation frequency of *S. coelicolor* when it was grown alone and when it was grown in competition with another *S. coelicolor* colony (mean alone: 0.056, range: 0.037–0.089; mean versus itself: 0.054, range: 0.037–0.083) (*U* = 79, *p* = 0.98). As expected, CFU declined as a consequence of resource competition between adjacent colonies, from 5.2 × 10^6^ of the control to 2.3 × 10^5^ when grown with another *S. coelicolor* (*U* = 160, *p* < 0.001; figure 4B). When *S. coelicolor* was grown adjacent to *S. ardus*, we also observed no change in mutation frequency when cells were plated simultaneously (mean: 0.058, range: 0.4–081 (*U* = 35, *p* = 0.70)). However, when we allowed *S. ardus* to grow for 2 days prior to plating *S. coelicolor*, mutation frequency was significantly increased (mean: 0.098, range: 0.081–0.117 (*U* = 2, *p* < 0.001); electronic supplementary material, figure S3), suggesting that secretion of mitomycin C increased over time. Similarly, mutation frequency was significantly increased when *S. coelicolor* was grown adjacent to *S. venezuelae* (mean: 0.089, range: 0.065–0.155 (*U* = 5, *p* < 0.001)). However, in contrast to the response to *S. ardus*, where mutation frequency already increased during simultaneous exposure to *S. venezuelae*, further exposure (for 2 days) had no additional effect (mean: 0.09, range: 0.064–0.127 (*U* = 54, *p* = 0.73)). Importantly, the CFU of *S. coelicolor* increased during competition with *S. ardus* and *S. venezuelae* (*U* = 14, *p* = 0.02; *U* = 11, *p* = 0.009) as compared with the control when it competed against itself. This suggests that the increased mutation frequency may provide a competitive advantage through increased antibiotic production.

## 4. Discussion

The linear chromosomes of *Streptomyces* have long been known to be unstable at the chromosome ends [19,21,22]. While the molecular mechanisms behind this phenomenon remain unclear, large chromosomal deletions are of considerable interest to industry because they potentially threaten the stability of antibiotic or enzyme production in industrial fermenters [34]. We recently studied the ecological and evolutionary consequences of chromosome instability in *S. coelicolor* and found that these mutations gave rise to a division of labour, whereby mutated cells overproduce antibiotics for the rest of the colony [17]. However, it remained unknown if the fraction of cells with mutations, which is analogous to the caste ratio of social insects, varied in different competitive conditions or stages of growth. Such plasticity would imply that mutation frequencies could be rapidly modified to increase the adaptability of strains during ecological challenge.

Here, we quantified mutation frequencies of hundreds of independent colonies through time and in different regions of the colony. Our results provide clear evidence that the mutation frequency increases as colonies grow and age (figure 1). Previous studies with unicellular bacteria have obtained similar results and hypothesized that this is due to the accumulation of e.g. reactive oxygen species (or other compounds) that are mutagenic or repress DNA repair [35,36]. However, an increased frequency of mutants does not necessarily imply that the basal mutation rate has also increased. ‘Allele surfing’, where mutants have an increased probability of fixation at the colony edge owing to increased resource access, can also lead to a similar spatial outcome without any change in the underlying mutation rate [30,31].

To distinguish these possibilities, we dissected colonies by separately analysing CFU and mutation frequencies from the middle and periphery of the colony (figure 2). As expected in growing colonies, CFU increased significantly on the edge of the colony between Day 7 and Day 14. However, in contrast to the predictions of ‘allele surfing’, mutation frequency only significantly increased in the middle of the colony, despite the fact that CFU remained unchanged in this region. We consider two, non-mutually exclusive, explanations for this pattern: (i) chromosome deletions occur in spores in the middle of the colony owing to exposure to mutagenic compounds (e.g. antibiotics) that are predominantly produced in this region; (ii) despite no change in CFU, there may be cryptic growth, chromosome replication and sporulation in the centre of the colony that are offset by germination of older spores, leading to no net change in CFU. Older studies relying on detailed microscopy confirm extensive ‘precocious’ germination within colonies, which is consistent with this second dynamic [37]. However, increased mutation frequencies in the colony centre would then require that newly formed spores have a higher mutation rate. *Streptomyces coelicolor* produces several developmentally regulated antibiotics, including the blue-pigmented actinorhodin and the red-pigmented undecylprodigiosin [14,15]. While actinorhodin has no known mutagenic functions, prodigiosin is a known mutagen whose expression is increased in aging hyphae [38–41]. These features make it a strong candidate for increasing mutation frequency in aging colonies and in a spatially restricted manner. We intend to test this directly in future studies using strains lacking prodigiosin and in environments where we can disrupt the spatial localization of this compound.

While intrinsic factors like prodigiosin might unavoidably increase mutation rates in aging colonies, external factors, e.g. cues derived from competitors or threats, could potentially adaptively increase mutation frequencies. Similar plastic responses are known from ants and wasps that respond to competitors by increasing the fraction of individuals that differentiate into soldiers. Antibiotics are an important ecologically relevant cue for *Streptomyces* given the role of these metabolites in interference competition [15,24,27,42]. Moreover, exposure to antibiotics, either with fixed concentrations in agar [43] or during co-culture with a competing strain [26,28], can induce antibiotic production. As shown in figure 3, several antibiotics significantly shifted the mutation frequency; however, we observed both increases and decreases in mutation frequency, even with antibiotics with similar modes of action on protein synthesis. This suggests that antibiotics may not be a general driver of increased mutation in *Streptomyces*, but rather that this depends on the specific mode of action. Additionally, these assays may be sensitive to the antibiotic concentration we used on our assays. Preliminary data with three antibiotics (novobiocin, ciprofloxacin and mitomycin C; V. Chopra 2021, unpublished data), as well as results from other groups [44–46], show a strong dose-dependence on mutation rates. Higher concentrations markedly increase mutations, but they also dramatically reduce CFU. It was for this latter reason that we chose to analyse strains at 0.25 MIC.

Because of potential limitations of using arbitrary concentrations of antibiotics, we carried out direct competition assays between species that naturally produce chloramphenicol and mitomycin C (figure 4). In both cases, we observed an increased mutation frequency, although this only occurred after giving *S. ardus* a 2 day head start to allow sufficient accumulation and diffusion of mitomycin C in the agar. More interestingly, this change coincided with increased spore production, suggesting that a higher fraction of mutant strains within colonies led to increased fitness. As yet, it remains unknown if this adaptive response is due to the change in mutation frequency, to antibiotic induction due to competition sensing or some combination of the two. Regardless, this supports the idea that increased antibiotic production in *S. coelicolor*, whether via mutation [17] or gene regulation [47], is phenotypically plastic and can respond adaptively to competition with other species.

The specific aim of this paper was to analyse intrinsic and extrinsic factors regulating mutation frequencies, and therefore the expression of division of labour, in *S. coelicolor*. We use mutation frequency as a proxy for social caste in analogy with socially determined castes in colonies of insects or other animals. While we believe our results uncover ecologically relevant plasticity in mutation rates in *S. coelicolor*, there are limitations to our conclusions and to the analogy with social insects. First, our determination of ‘caste’ is limited to strains with obviously aberrant morphology, which may lead to a systematic underestimate of caste ratios if some fraction of chromosome deletions do not cause phenotypic changes. Second, as yet, the causal link between increased mutation frequency and increased competitiveness remains unclear. We know that a higher mutant fraction within colonies leads to higher antibiotic production [3], but further work will be required to determine how this results in increased CFU during competitive interactions. Finally, even though the present work shows that the caste ratio in *Streptomyces* is ‘flexible’, it remains unknown if it can be optimized to ensure the greatest benefit from antibiotic production (owing to increased mutation rate) at the lowest cost to the colony. Optimal caste ratios have been extensively studied in social insects [6], but there is no comparable theoretical literature on bacterial divisions of labour. Addressing these experimental and theoretical limitations remains an important objective of future work.

## Data Availability

Data are available from the Dryad Digital Repository [48]. Supplementary material is available online [49].
